# Predictive role of neostromal CD10 expression in breast cancer patients treated with neoadjuvant chemotherapy

**DOI:** 10.3389/pore.2022.1610598

**Published:** 2023-01-05

**Authors:** Orsolya Olah, Edit Majlat, Renata Koszo, Zoltan Vereb, Andras Voros

**Affiliations:** ^1^ Department of Pathology, School of Medicine, University of Szeged, Szeged, Hungary; ^2^ Department of Oncotherapy, School of Medicine, University of Szeged, Szeged, Hungary; ^3^ Department of Dermatology and Allergology, School of Medicine, University of Szeged, Szeged, Hungary

**Keywords:** advanced breast cancer, predictive factors, CD10, neoadjuvant therapy, neostroma

## Abstract

**Background:** The therapeutic strategy of invasive breast cancer is based on routine histopathological markers (estrogen-, progesterone receptor, HER2, Ki67) routinely evaluated in tumor cells. However, the assessment of cancer stroma could influence therapeutic strategies. Studies have shown that stromal expression of CD10, a zinc-dependent metalloproteinase, is associated with biological aggressiveness of the tumor. In the present retrospective study, we aimed to evaluate stromal CD10 expression and association between CD10 expression and response to neoadjuvant chemotherapy in invasive breast cancer.

**Methods:** CD10 immunohistochemistry was performed on core biopsies taken before the neoadjuvant therapy. Stromal CD10 expression was determined and compared with well-known predictive and prognostic tissue markers as well as with the following groups defined according to the degree of tumor response: no regression, partial regression, and complete regression.

**Results:** A total of 60 locally advanced invasive breast carcinomas of “no special type” were included. The proportion of CD10 positive tumors was significantly higher in the “no regression” group compared to “complete regression” group (*p* = 0.000). Stromal CD10 expression was found to be significantly associated with decrease in response to neoadjuvant chemotherapy. According to CD10 expression we did not find any difference in hormone receptor status, Ki67, tumor grade or neostromal area.

**Conclusion:** Our data suggest that CD10 expression can serve as a predictive marker of the effect of neoadjuvant chemotherapy in breast cancer patients. Therefore, its inclusion into the routine assessment of biopsies to tailor tumor-specific therapeutic strategies merits consideration.

## Introduction

Although early breast cancer detection programs are well organized in developed countries, a number of patients remain hidden until developing late-stage disease. In locally advanced cases, primary systemic chemotherapy is often indicated, and the choice of treatment is influenced by the evaluation of routine prognostic and predictive factors. Unfortunately, proper quantification of estrogen- and progesterone receptors (ER and PR), human epidermal growth factor receptor-2 (HER2/Neu) and proliferation markers are insufficient to predict chemosensitivity of some breast tumors. In these cases, despite the high proliferation and receptor statuses, chemotherapy remains unsuccessful, and the fast progression is associated with recurrence and high mortality. The identification of these cases during routine pathological examination of biopsy specimens could be especially useful in planning the oncotherapeutic strategy for proper patient management.

Recently, not only the epithelial component of breast cancers, but also their microenvironment, particularly the neostroma with immune cells came into focus. Tissue microenvironment has a vital role in promoting and controlling the development and dissemination of malignant tumors (1, 2). Particularly, tumor-infiltrating lymphocytes are of therapeutic relevance, in case of high PD-L1 expression.

Carcinoma-associated fibroblasts (CAFs) are a major constituent of the tumor stroma and represent a heterogenous population of activated fibroblasts (3). CAFs play a crucial role in tumor development through promotion of tumor progression and maintenance of chemoresistance (4). Special subtypes of CAFs with different biologic role are identified *in vitro* and *ex vivo*, and this is based on their intracellular cytokine expression. There are also attempts to identify and characterize CAFs on routine samples, as this may clarify their role in tumor development (5).

CD10 is a 90–110-kDa cell surface zinc-dependent metalloprotease that is normally expressed by the epithelial cells of diverse tissues, including prostate, colon, liver, stomach and apocrine breast lesions, but is also present in endometrial stroma (6–9). The detection of CD10 is a useful tool in numerous pathological entities, such as acute lymphomas and non-malignant tumors of the breast (10). As many of the matrix metalloproteinases (MMPs), CD10 is accountable for degradation of extracellular matrix components. It also regulates the biological activities of various peptide substrates by lowering the local concentrations available for receptor binding (11). It has also been proposed that CD10 overproduction appears in tumor neostroma, and this may contribute to tumor development and progression by degrading extracellular matrix and giving way to local invasion (3). Studies have also shown that in other types of tumors the expression of CD10 in stromal cells is associated with higher grade and agressive biological behavior (12, 13).

According to recent studies, CD10 has a possible role in the progression of breast cancer, too, since this marker is found to be expressed by CAFs in tumors with faster progression (5). It is also reported, that tumors with higher number of CD10 positive CAFs are less chemosensitive *in vitro* (5). Since neoadjuvant chemotherapy is the most used therapy in locally advanced breast cancer (LABC), patients with chemoresistance or only with minor chemosensitivity can have an extended time to curative surgery.

We aimed to examine CD10 expression on initial routine diagnostic core biopsies from patients with newly discovered LABC, and correlate this with tumor regression achieved during neoadjuvant chemotherapy.

## Materials and methods

This retrospective study was conducted in the Department of Pathology of the University of Szeged. It was approved by the Ethical Committee of University of Szeged (265/18-SZTE).

### Study population and samples

The retrospective cohort included cases of LABC diagnosed at the University of Szeged between 2010 and 2018 and treated with neoadjuvant chemotherapy. For inclusion, the tumor had to fulfill the criteria of LABC defined by the 4th Hungarian Breast Cancer Consensus Conference; the tumor had to be locally advanced, primary, with or without multifocality and/or lymph node metastasis without distant metastasis (Stage IIb to Stage III) (14). All patients received anthracycline based chemotherapy followed by surgical treatment.

The initial core needle biopsy sample and the surgically removed tissue sample with the related histologic reports were provided by the Department of Pathology. The predictive and prognostic markers were obtained during the routine histopathologic examination according to international guidelines and the 4th Hungarian Breast Cancer Consensus Conference, and included the ER-, PR status and Ki67 proliferation fraction (labelling index). HER2 protein expression was determined *via* immunohistochemistry, in the case of equivocal expression *in situ* hybridization was also performed. Tumor-infiltrating lymphocytes (TIL) was defined according to the protocol established in 2014 by an International TILs Working Group and cases exceeding 5% were considered positive (15). The evaluation of these assays followed internationally accepted recommendations (16, 17) in keeping with national guidelines (18). All core biopsy samples contained more than 70% neoplastic area.

The initial tumor size was determined according to the radiologic finding made at the time of core biopsy or staging examination prior to the neoadjuvant treatment.

The experimental groups were determined according to the regression grade assessed on surgical specimens following neoadjuvant chemotherapy. The regression grade was also retrieved from the pathological reports and was reported according to the Hungarian Breast Cancer Diagnostic guidelines (18) based on the European guidelines (19). According to regression grades, three groups were defined: 1) no regression (TR3), 2) partial regression (TR2), and 3) complete regression (TR1).

### Immunohistochemistry and evaluation

CD10 immunohistochemistry was performed on 4 μm thick sections of the core needle biopsy samples with an automated method (Leica Bond autostainer, Wetzlar, Germany). The primary monoclonal antibody (CellMarque, Rocklin, California, United States) was used with a dilution of 1:50 (incubation time 20 min) and was applied after antigen retrieval in ER2 (pH9).

**FIGURE 1:**
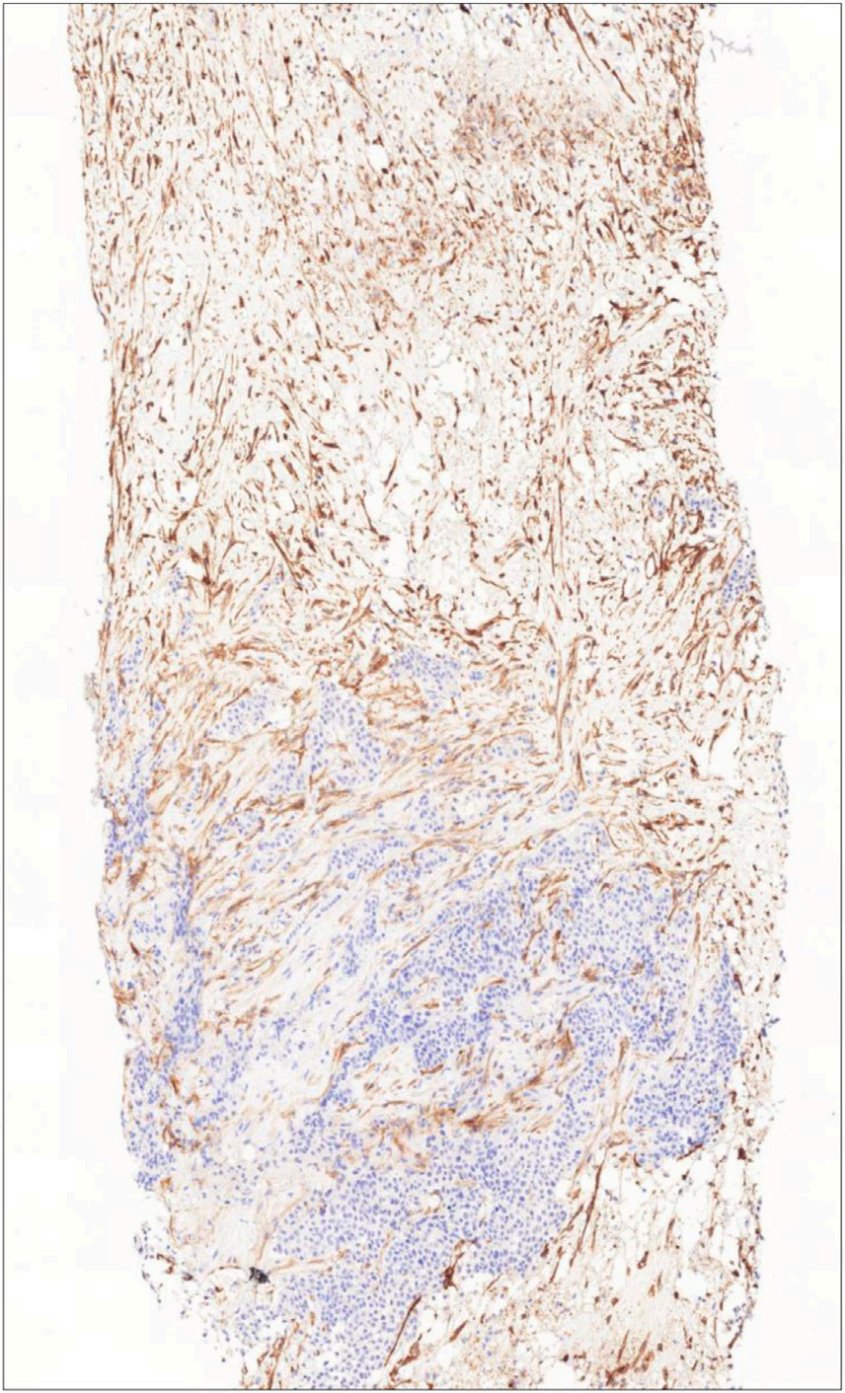
Intensive CD10 expression of stromal cells representing the whole stromal area (CD10 immunohistochemistry, 10X).

CD10 expression in the tumor stroma (both cellular elements and extracellular matrix) was assessed qualitatively and quantitatively. Qualitative analysis was conducted by two independent pathologists (OO and VA) who were unaware of the response of the tumors to primary systemic therapy. The samples showing stromal expression greater than 10% were considered positive, following the methodology of Iwaya et al (20) (Figure 1). In case of disagreement the case was reviewed by both investigators and consensus was made.

Quantitative analysis was performed on digitalized slides (Pannoramic Midi slide scanner, 3DHistech, Budapest, Hungary). Overwiev pictures were made on ×10 magnification. The digital evaluation program was treated to recognize and distinguish the epithelial component from the stroma by labelling different components. The process was controlled by pathologist (OO). In the analysis phase the automatic area selection was continuously monitored and, if necessary, corrected it manually to avoid epithelial expression involvement. The pixels representing neostroma were conwerted to black and white pixels according to brown color density reflecting the diaminobenzidine chromogen of the immunohistochemistry, also normalized to background brown staining. Black represented the immunostained areas, while white the unstained areas. The proportion of the stained (black) area was derived from the area stained and its relation to the total neostromal area (Pannoramic QuantCenter modules, 3DHistech, Budapest, Hungary).

### High content imaging system and image analysis

The Olympus IX83 microscope based high content screening platform (Olympus, Tokyo, Japan) was used for image acquisition and fully automated analysis of all images taken from all specimens. To get a higher X/Y resolution than would be possible with a single field of view, stitched images of the full tissue specimens were made. Automated stitching alignment was carried out with meander scanpath and alpha (linear) blending.

Super resolution images were analyzed with the automated method of the CellSense software (Olympus, Tokyo, Japan).

### Statistical analysis

Statistical analyses were performed using the SigmaPlot 12.0 software (Systat Software Inc., San Jose, CA, United States). Data were expressed as mean ± standard error of the mean (SEM) derived from at least three independent measurements. The clinical data were analyzed with chi-square test. The stromal area as a proportion of total tumor area and both qualitative and quantitative measurement of CD10 expression were examined using the t test for independent samples (two groups) or ANOVA (more than two groups); *p* < 0.05 was considered statistically significant. In cases ANOVA showed significance Bonferroni *post hoc* test was used to compare groups.

## Results

### Qualitative CD10 expression

A total of 60 patients were included with 20 cases in each group. Their mean (±standard deviation, SD) age was 53.5 ± 8.6 years. The remarkable clinical data distribution among groups is listed in Table 1 and the protocol of neoadjuvant oncotherapy in detail is listed in the Supplementary Table S1.

**TABLE 1:** Distribution of clinical data among groups.

		Complete regression	Partial regression	No regression	*p* value
Molecular type	Luminal B	9	9	10	0.9965
	HER2	4	4	4	
	TNBC	7	7	6	
Stage	II	8	9	10	0.8170
	III	12	11	10	
Grade	II	2	4	3	0.6756
	III	18	16	17	
Tumor size	Mean	45.18	38.33	29.58	**0.0448**
	SD	18.40	26.25	9.51	
	n	20	20	20	

The bold value means that the value is significant.

**FIGURE 2:**
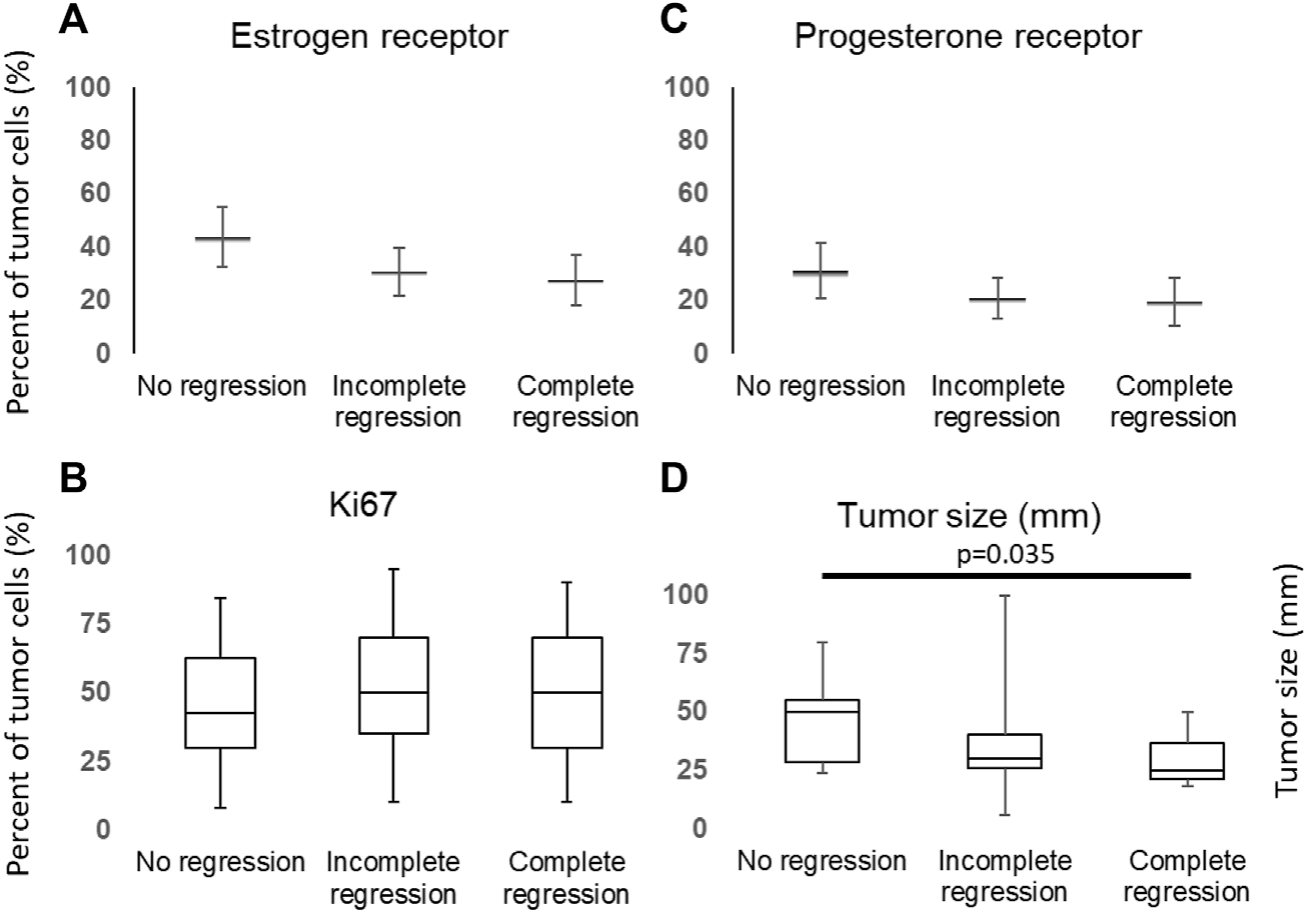
Distribution of ER **(A)**, PR **(C)**, Ki67 **(B)** and tumor size **(D)** among cases and groups.

There were no statistical differences between the regression groups in terms of mean proportion of ER positive tumor cells (43.75 ± 11.18, 30.55 ± 9.09, and 27.56 ± 9.54, respectively), PR positive tumor cells (31.25 ± 10.28, 20.83 ± 7.73, and 19.4 ± 8.80, respectively) and expression of Ki67 (50.63 ± 5.95, 54.69 ± 5.64, and 53.21 ± 7.33, respectively) (Figure 2).

We also have found significant differences between initial tumor sizes (*p* = 0.0448), the median of initial tumor size was significantly lower in the complete regression group than in the group showed no regression (pairvise comparison: *p* = 0.035, Figure 2D), although the tumor size did not differ regarding CD10 expression (Supplementary Figure S1).

There were also no significant differences in the proportion of HER2 positive cases (Table 2). The ratio of (stromal) tumor infiltrating lymphocytes (TILs) was lower than 5% in majority of cases; furthermore, there was no difference in the mean proportion of TILs among the groups (Table 2).

**TABLE 2:** Distribution of HER2 and TIL positive (>5%) and negative (<5%) cases among groups.

Group		TIL	HER2
No regression	Positive	2	5
	Negative	18	14
Partial regression	Positive	3	4
	Negative	17	16
Complete regression	Positive	4	6
	Negative	16	15

**FIGURE 3:**
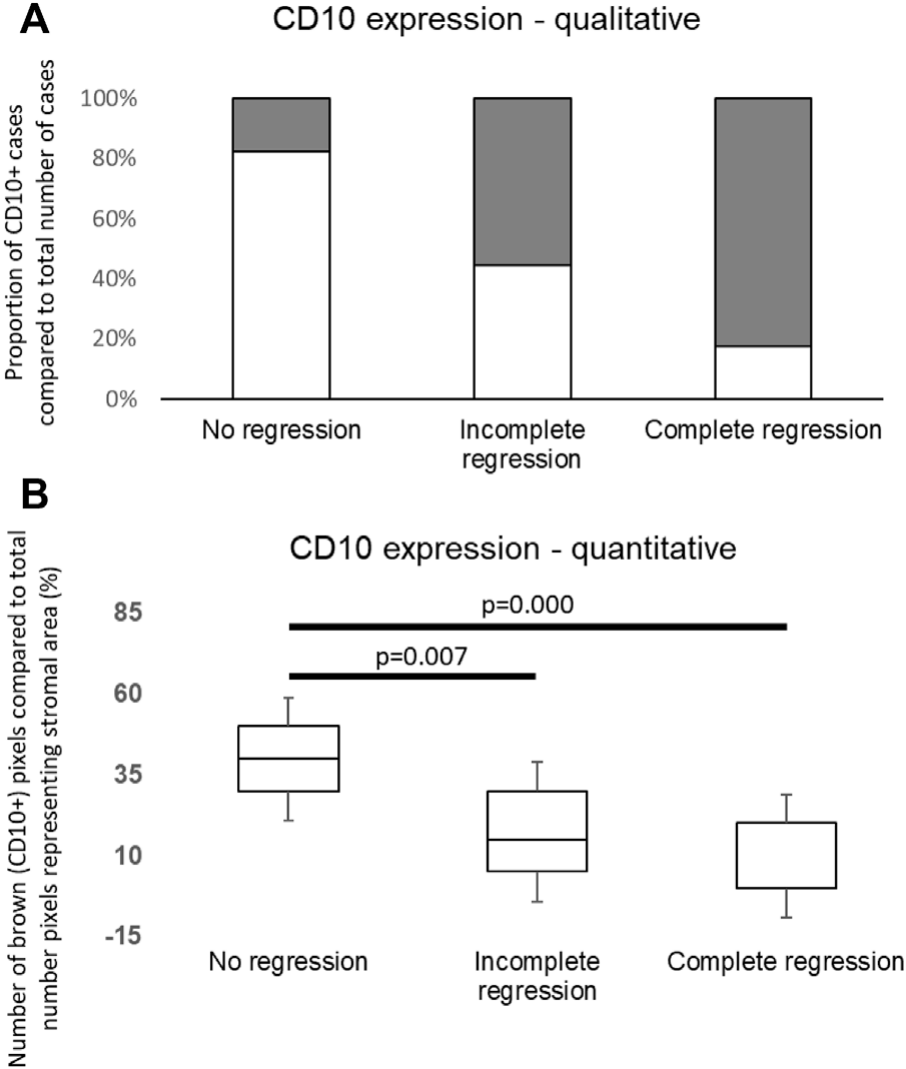
**(A)** Distribution of CD10 positive and negative cases in groups. The white columns are indicating positive (>10% stromal CD10 immunolabeling) cases. The number of CD10 positive cases was significantly higher in the non-responding group to neoadjuvant chemotherapy (*p* = 0.006). **(B)** Quantitative evaluation of CD10 expression showed significant differences among distinct groups similarly to qualitative data.

CD10 signals were found in both the epithelial and stromal components, nevertheless only neostromal expression was considered. In order to determine applicability of CD10 immunohistochemistry in routine histopathologic work, a qualitative “eye-balling” evaluation with a 10% cut-off value was made. The investigators gave concordant results except one case in the complete regression group that was later considered positive according to their consensus. This qualitative evaluation of CD10 expression suggested significantly less CD10 positive cases among tumors showing complete regression. In parallel, approximately half of the cases in the partial regression group and most of the cases in the no regression group were CD10 positive (Figure 3A).

To determine the expression of CD10 in neostroma more precisely, quantitative measurements were made. Our quantitative evaluation with both evaluation methods (Panoramic Quant Centre and Olympus CellSense) showed similar results to those of the qualitative estimations; the ratio of area showing immunopositivity for CD10 was significantly different in the defined subgroups (*p* = 0.019) with significant difference between the “no regression” and “incomplete regression,” also “no regression” and “complete regression” groups (*p* = 0.007 and *p* = 0.000, respectively, Figure 3B).

According to our measurements the specificity of CD10 immunohistochemistry was 82%, while sensitivity was 83%.

**FIGURE 4:**
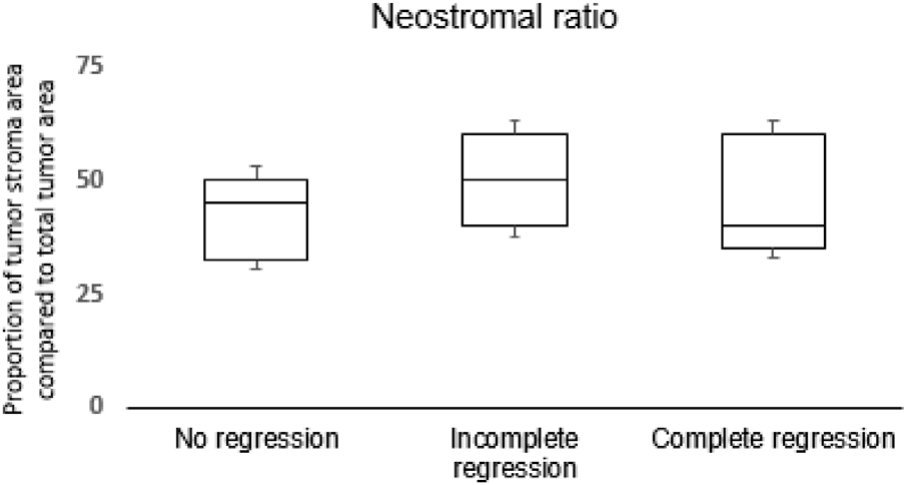
Distribution of neostromal area in proportion of the total tumor area among groups.

To exclude the variability in desmoplastic reaction, we evaluated the proportion of neostroma and total tumor area, and this showed no differences among the three groups (Figure 4).

We evaluated the routine predictive and prognostic parameters according to CD10 positivity. Although the number of cases was not sufficient for proper correlation studies there were no significant differences in estrogen-, progesterone receptor, Ki67 expression and HER2 status, histologic grade and neostromal ratio between CD10 positive and negative cases (Table 3). Since we did not notice differences in these parameters depending on the CD10 status we considered CD10 expression independent.

**TABLE 3:** Expression of routine prognostic markers depending on CD10 positivity.

			CD10 negative	CD10 positive	*p*
Estrogen receptor	Mean		32.92	24.83	0.424
	SD		38.73	39.04	
	n		29	31	
Progesterone receptor	Mean		13.33	23.74	0.231
	SD		29.14	36.72	
	n		29	31	
Ki67	Mean		49.68	47.72	0.772
	SD		30.46	21.09	
	n		29	31	
HER2	n		7	8	0.881
Grade	II	n	4	5	0.800
	III	n	25	26	
Neostromal ratio	Mean		46.11	40.83	0.148
	SD		15.03	12.81	
	n		29	31	

## Discussion

Although breast cancer is an epithelial malignancy originating from cells of the terminal ductal lobular unit, it is well documented that tumor microenvironment has also significant role in its biological behavior. It has been proved that tissue microenvironment as well as the tumor neostroma play a key role in controlling cell survival, proliferation, migration, and differentiation (1, 2, 21). CAFs as a special cell type of the neostroma, can promote cancer progression by regulating cancer stem cells and chemoresistance (22).

The close bilateral molecular cooperation between the epithelial and stromal cells is disrupted by several factors/expressed proteins that are secreted by either the tumor cells or the stromal cells modulated by the epithelial component of cancer (23, 24). Members of the MMP group are examples of these factors and play a key role in tumor progression, invasion and metastasis (25, 26). Up-regulation of extracellular matrix gene expression and elevated MMP activities correlate with poor patient prognosis (26). High expression of estrogen receptors is also associated with enhanced activity of MMP-2, while high expression of progesterone receptor is correlated with low TIMP-1 protein (tissue inhibitor of MMPs) levels that promotes matrix destruction (25). The MMP expression also plays an important role in the production of TGF-β (tissue growth factor-β) secreted by the CAFs and this promotes tumorigenesis, angiogenesis, immunosuppression and tumor progression (26). The breakdown products of the matrix components produced by MMP activity together with growth factors (insulin like growth factors) have chemotactic activity thus help in tumor cell migration through the extracellular matrix (27).

CD10 is a membrane-bound zinc-dependent metalloproteinase with endopeptidase activity, which regulates the physiological action of various peptides by lowering their extracellular concentration available for receptor binding (10, 28). CD10 has widespread functions in maintaining early progenitor population in the human mammary lineage thus preventing the unchecked proliferation in mammary stem cells under physiologic conditions (29).

CD10 has also roles in breast cancer formation. Maguer-Satta et al have shown that an early oncogenic event in stem cells modulates the expression of the CD10enzyme in the altered cells or even in the neighboring cellular environment resulting in a decrease of CD10 function and a commitment in neoplastic lineage (10). It has also been shown that upregulation of mutated CD10 enzymatic activity could lead to accumulation of cleaved peptides that inhibit cell differentiation and maintain the state of cancer stemness (28).

There are some previous studies concluding that stromal expression of CD10 is an obvious negative prognostic factor of breast cancer. It is correlated with higher grade, node positivity, increased Ki67 index, positive HER2 status and poorer prognosis supplemented with the antiapoptotic effect that plays role in reduced chemosensitivity (12, 20, 30-34). Diem Vo et al. concluded that CD10 expression in stroma may function as a powerful prognostic factor for invasive breast cancer disease-free survival rates, and in predicting potential recurrence (35). Desmedt at al. Highlighted the importance of CD10^+^ cells in breast cancer prognosis and efficacy of chemotherapy. This was principally seen when they characterized CD10^+^ cells isolated from tumor vs. normal breast containing cell cultures (36).

Our retrospective study has shown that the presence of CD10 in the neostromal component of breast cancer is commonly associated with resistance to neoadjuvant treatment in LABC. These findings are in partial concordance with preliminary findings of Thomas et al. They have found that stromal CD10 expression in breast cancer is not static and changes over time in breast cancers treated with neoadjuvant anthracycline based chemotherapy. Stability or decrease in CD10 expression correlates with complete or partial clinical response, while an increase in CD10 expression appears to correlate with poor clinical response. They also mentioned that stromal CD10 expression and its changes with chemotherapy may have a prognostic significance, they should be documented in breast cancer patients before and after chemotherapy (30). Jana SH and al reported poorer prognosis associated with CD10 expression although they have not assessed chemoresistance separately (31).

We have also shown that a qualitative analysis of CD10 expression with a 10% cut-off gives similar results to the quantitative analysis, and this facilitates its application in a routine histopathological diagnostic setting. Although the qualitative analysis of CD10 immunohistochemistry is easy to implement there are several limitations. The qualitative analysis of any staining shows variable reproducibility; thus, the evaluation guidelines have to be clearly defined. Therapeutic strategies are limited to neoadjuvant therapies or extended surgical removal in LABC. The most preferred strategy is neoadjuvant chemotherapy with or without targeted treatment, which is planned according to routine predictive and prognostic markers tested during histopathologic examination. Despite onco-radio-pathologic correlation and precise therapeutic planning, chemotherapy fails in a number of cases, and this results in a delay in surgical treatment. Prediction of chemosensitivity could be an effective method to refine oncotherapeutic strategy allowing personalized breast cancer patient management. The CD10^+^ CAF type described by Su S et al. also gives the opportunity for targeting neostromal background of cancer in cases non-responding to conventional chemotherapeutic agents (5).

Albeit there are studies showing correlation between histology subgroups and CD10 expression our study focused on mixed population of LABC to investigate the theoretical usability of CD10 immunohistochemistry in predicton of chemosensitivity. For precise determination of LABC subgroup suitable for chemosensitivity prediction with CD10 immunohistochemisty further studies have to be made.

Despite limitations of the study our results show correlation between CD10 expression and chemosensitivity. This emphasizes the potential of CD10 immunohistochemistry in routine histologic examination as a predictive marker. CD10 did not show unequivocal concordance with the well-known predictive and prognostic markers (ER, PR, HER2 and Ki67). CD10 expression was independent from tumor size, too, although tumors showing complete remission following neoadjuvant chemotherapy had significantly smaller initial tumor sizes. These findings suggest that stromal CD10 could be a predictive marker of treatment efficacy and chemosensitivity that warrants further studies.

## Conclusion

In conclusion, we analyzed the expression of CD10 in a series of pre-treatment core biopsies taken from advanced breast cancer patients candidate for neoadjuvant chemotherapy. Our results indicate that the higher the stromal CD10 expression on pre-treatment core biopsies, the worse the regression after neoadjuvant chemotherapy. We also found stromal CD10 to be independent from other well-known prognostic and/or predictive markers like ER, PR, HER2 and Ki67 in the neoadjuvant setting. These findings highlight a role of CD10, as a possible independent predictive marker of the possible efficacy of neoadjuvant chemotherapy in case of breast cancer. We also propose that CD10, despite some technical limitations mentioned above, could implement the conventional immunohistochemistry in the course of planning neoadjuvant chemotherapy. and requires further investigation.

## Data Availability

The original contributions presented in the study are included in the article/Supplementary Material, further inquiries can be directed to the corresponding author.
