# Supplementary material for: Predictive role of neostromal CD10 expression in breast cancer patients treated with neoadjuvant chemotherapy
Source: Pathol Oncol Res. 2023 Jan 5;28:1610598. doi: 10.3389/pore.2022.1610598 (PMC9849231; doi:10.3389/pore.2022.1610598)
Supplement: Supplementary file 1 [file Presentation1.PPTX]

## Slide 1
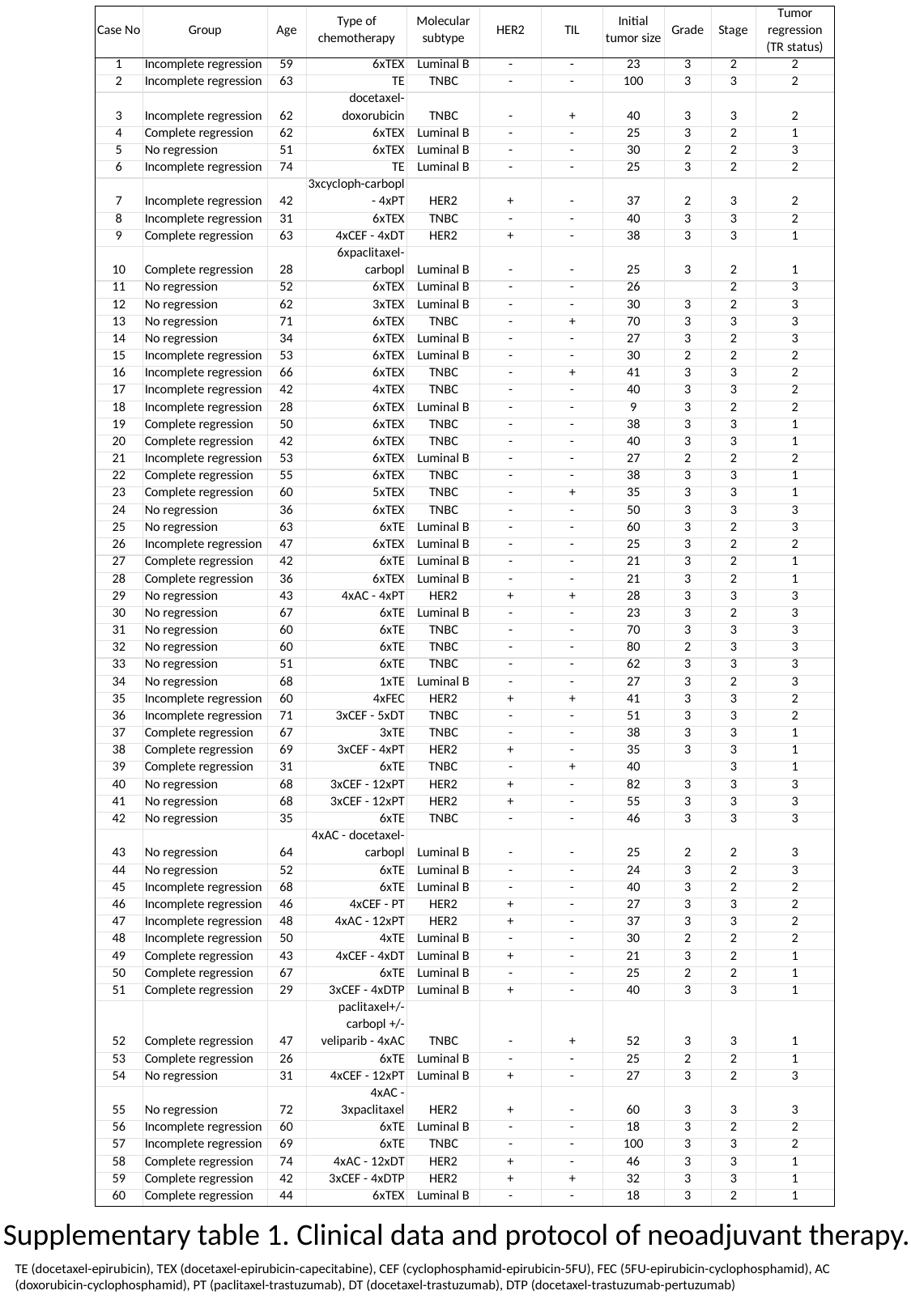

Supplementary table 1. Clinical data and protocol of neoadjuvant therapy.
TE (docetaxel-epirubicin), TEX (docetaxel-epirubicin-capecitabine), CEF (cyclophosphamid-epirubicin-5FU), FEC (5FU-epirubicin-cyclophosphamid), AC (doxorubicin-cyclophosphamid), PT (paclitaxel-trastuzumab), DT (docetaxel-trastuzumab), DTP (docetaxel-trastuzumab-pertuzumab)

## Slide 2
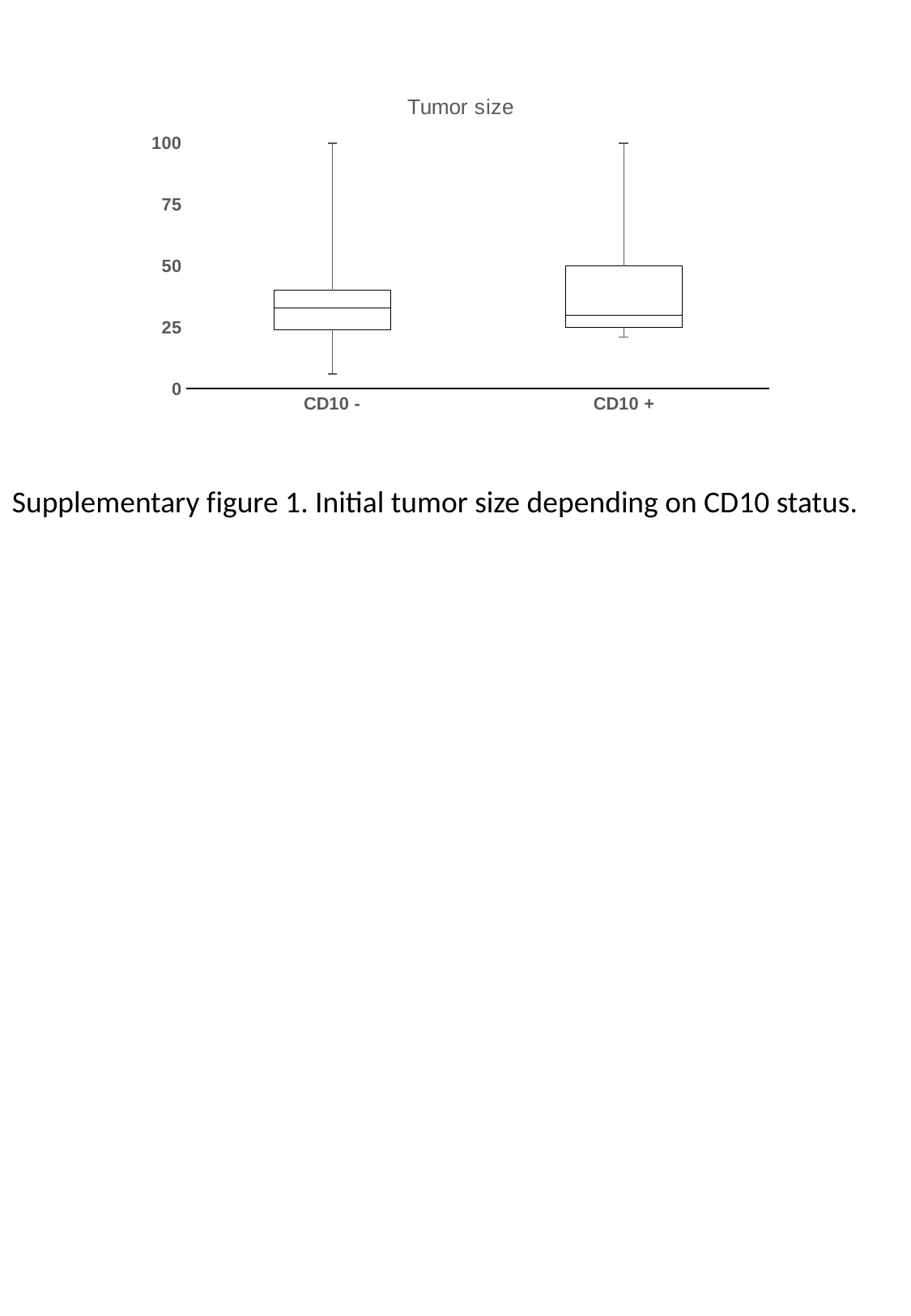

### Chart: Tumor size
| Category | Q1 | median-Q1 | Q3-median |
|---|---|---|---|
| CD10 - | 24.0 | 9.0 | 7.0 |
| CD10 + | 25.0 | 5.0 | 20.0 |Supplementary figure 1. Initial tumor size depending on CD10 status.
